# Towards Personalised Nutrition in Major Orthopaedic Surgery: Elements of Care Process

**DOI:** 10.3390/nu17040700

**Published:** 2025-02-16

**Authors:** Matteo Briguglio, Thomas W. Wainwright

**Affiliations:** 1 IRCCS Ospedale Galeazzi—Sant’Ambrogio, Laboratory of Nutritional Sciences, 20157 Milan, Italy; 2 Orthopaedic Research Institute, Bournemouth University, Bournemouth BH12 5BB, UK; 3 University Hospitals Dorset, NHS Foundation Trust, Bournemouth BH7 7DW, UK; 4 Lanzhou University, Lanzhou 730070, China

**Keywords:** orthopaedic procedures, hip replacement arthroplasty, arthroplasty, knee replacement, enhanced postsurgical recovery, nutrition sciences, diet, food, and nutrition, precision medicine, food supplement

## Abstract

With the spread of enhanced recovery protocols, the management of the perioperative pathway of patients undergoing major orthopaedic surgery has been harmonised to these international standards. A natural evolution of the enhanced recovery framework is to integrate personalised pathways of care for those with unique needs, thus addressing inter-individual differences. Personalised nutrition is the practice of attributing a personal imprint to the perioperative nutritional support and has the potential to ensure more effective and equitable care for those patients who may require more than standard support. The authors of this opinion article review each important element of personalisation with respect to their coverage of what is important in the perioperative care of major orthopaedic procedures such as hip and knee replacement.

## 1. Background

The enhanced recovery approach has represented a transformative milestone in perioperative medicine. Before enhanced recovery, the management of patients undergoing major orthopaedic surgery was often characterised by inconsistencies both between institutions and within the same hospital. These variations were often unintentional, stemming from poorly defined processes, entrenched traditions, and outdated dogma. Enhanced recovery has harmonised practices across settings, providing guidance with standardised protocols that are evidence-based and safe [1]. This has reduced unwarranted variations in care processes and achieved improved outcomes. Despite these successes, there remain opportunities to refine pathways as long as science progresses. Personalised nutrition care is one such opportunity that holds great promise in further reducing complications, speeding up recovery, making the treatment path more patient-responsive, and improving long-term quality of life [2]. But what exactly is meant by “personalised nutrition”?

## 2. Definition of Personalised Nutrition

The adjective “personalised” can be regarded as an umbrella term. It is often used interchangeably with individualised, tailored, optimal, precision, or customised nutrition [2]. However, these terms have subtle semantic differences worth clarifying (Table 1). In this article, the term personalisation will be preferred over the other terms.

## 3. Elements of Personalised Nutrition for Orthopaedic Surgery Practice

Adopting a healthy and balanced diet that meets energy and nutrient requirements is important to optimise nutrition care before and after undergoing a major orthopaedic surgery, such as joint (total hip or knee replacement) and spine (e.g., lumbar interbody fusion) procedures. While general healthy eating guidelines offer a basic standard of care quality for most patients [3], they may fall short for individuals with specific needs. For instance, patients with excess body fat may benefit from reducing energy intakes, those with sarcopenia require precise control of protein and energy intakes, and patients with iron deficiency anaemia need careful monitoring of the intakes of iron and vitamin cofactors [4]. A personalised dietary approach addresses these varied requirements, offering an opportunity to further enhance healthcare quality for these specific patient subgroups. Though numerous elements ought to be considered, most are often overlooked in clinical practice. Below, the critical factors that should guide personalised nutrition are outlined, encompassing medical and lifestyle aspects, patient acceptability, and operational elements.

### 3.1. Body Measures, Compartment Estimates, Age, and Sex

These basic elements form the foundation of basal need calculations, determining daily energy requirements, the proportions of macronutrients like carbohydrates and proteins, and hydration [5]. Body-fat mass, estimated through bioelectrical impedance analysis or the more precise computed tomography/magnetic resonance imaging, is required for the diagnosis of malnutrition phenotypes, like sarcopenic obesity, and it could prove to be a more appropriate yardstick than body weight for fine-tuning of basal requirements. Beyond quantity, diet quality is essential, focusing on nutrient sources and dietary patterns (e.g., the Mediterranean diet). The definition of both the quantity and quality of nutrient sources based on reference values for sex and age is the starting point for a personalised dietary intervention.

### 3.2. Activity, Rest, and Sleep

Energy expenditure varies significantly between sleep and waking. Several factors, encompassing the type and intensity of physical activity, the thermic effect of food, and mass-specific metabolic rates of organs and tissues, play a critical role. Heart rate is one of the physiological parameters that most dictates average daily metabolic rate, while organ metabolism is essentially associated with the variation of energy demands throughout the life course and between sexes [6]. Similarly to body compartment approximations, there are methods more appropriate than predictive equations to better personalise energy expenditure estimation, such as indirect calorimetry for the bedridden patient or doubly labelled water for the free-living individual. Patients undergoing major orthopaedic surgery may limit movements due to pain before surgery, while on the contrary, they are required to align to exercise therapy goals after surgery [7]. Daily activity factors and diet-induced caloric demand multiply the energy expenditure at rest, representing important components when planning a personalised diet.

### 3.3. Disease Status and Surgical Trauma

When dealing with patients with any spell of ill health or medical condition, a personalised approach to nutrition intervention is required. Food administration is planned based on the oral, enteral, or parenteral route. Examples of disease-derived adjustments of the medical nutrition therapy are food abstention in food allergy, eating support in dysphagia, a repletion prescription to correct iron-deficiency anaemia, carbohydrate counting for diabetes mellitus, or therapeutic diets for renal and liver disease. Similarly, it is known that surgery triggers inflammatory and hormonal responses, temporarily altering metabolic functions. Postoperative nutrition should also be personalised to these injury-derived changes, for example, with increased protein intakes than usual to mitigate catabolism [8] or higher calories in case of fever.

### 3.4. Medications

Drugs can interfere with food components at various levels (Table 2) altering the nutritional efficiency of foods (Table 3).

A well-known example is the proton pump inhibitor, which interferes with dietary iron absorption. Food–drug interactions are bidirectional, as food is also known to alter the bioavailability of medicines, especially for those drug molecules whose absorption is pH- and site-dependent (e.g., via delay of gastric emptying, stimulation of bile flow, change in gastrointestinal pH, increase in splanchnic blood flow, and change in luminal metabolism). Two well-known examples are dietary lipids, which by slowing gastrointestinal motility increase the contact time of the drug with the tissue (e.g., risk of toxicities of chemotherapeutics in high-fat meals), and dietary proteins that directly interact with dopamine precursors like levodopa (i.e., reduced drug efficacy in Parkinson’s disease) [10]. A diet personalised on medications goes beyond indications about a full or empty stomach administration and should aim at preventing drug-derived nutrient deficiencies and avoiding pharmacological decompensation.

**Table 3:** Glossary of terms used when referring to nutritional efficiency [11].

Term	Definition
Food-matrix effect	Interactions with the food matrix that influence the free fraction of the compound in the food product.
Nutritional bioaccessibility	Fraction of the compound accessible after ingestion, gastrointestinal passage, and metabolism in the gut lumen.
Nutritional biodigestibility	Fraction of the compound that enters the circulation.
Nutritional bioactivity	Fraction of the compound that is active after assimilation into the target tissue.
Nutritional bioavailability	Fraction of ingested food compound that manifests its bioactivity at the biological target.

### 3.5. Timing

This element incorporates various concepts, including the time scheduling of each meal, when to consume a certain dish (e.g., vegetables at the beginning of the meal to promote early satiety), and the provision of dietary changes within the 24 h cycle and beyond [3]. Examples of time indications include the avoidance of high-fat meals in the evening for better weight control (circadian period) and the planning of a complementary iron therapy during menstruation (circa-monthly period) or of vitamin D in winter (circannual period). Furthermore, the time element deals with any dietary progression based on the life stages (e.g., children versus older adults) or due to the worsening of symptoms that may require transitory indications (e.g., temporary avoidance of lactose during diarrhoea and of fibre during flare-ups of Crohn’s disease).

### 3.6. Meal Preparation

Whether the patient is prescribed a daily or weekly food plan to follow, a personalisation of meal preparation methods can make the difference in terms of food safety and nutritional efficiency. Kitchen hygiene and cooking practices are known to establish a safe storage, texture (e.g., liquidised/thin purée, thick purée/soft and smooth, finely minced, modified/normal, regular) that is essential in swallowing disorders, nutrient content, bioavailability, and metabolic effects. For instance, cooking modifies the bioaccessibility of proteins and the bioactivity of vitamins [11], and the macronutrient composition of the meal influences the glycaemic responses [12].

### 3.7. Preferences and Food Liking

Eating behaviour is primarily driven by individual preferences and cultural influences. Food-liking in particular, which is the pleasantness of taste of food in the mouth, has its roots in genetics and biology [13] and is influenced by environmental factors like mass communication [14]. Exploring these elements of preference or palatability -pleasantness of eating that can be boosted with spices and aromatic herbs- can help clinical dietitians to direct patient’s food choices towards healthier options that are more likely to be enjoyed and adhered to.

### 3.8. Ethnicity and Religious Beliefs

Food choices are closely tied to the several attributes of heritage passed down through generations. Ethnicity is a proxy that can shape alcohol preference, liking for protein-rich foods, or aversion to pork [15]. Religious/spiritual beliefs, especially those carrying norms and taboos on food (e.g., abstention of pork in Islam, fish consumption during lean days for Christianity), can also shape dietary patterns [16] and even adversely affect a patient’s health if not addressed properly (e.g., fasting from dawn to sunset during Ramadan) [17]. Recognising these attributes ensures dietary recommendations respect patient values while maintaining nutritional adequacy.

### 3.9. Economic Resources

A healthy diet is unaffordable when the cost of purchasing the least expensive locally available foods to meet nutritional requirements exceeds the income available for food [18]. A patient with a low disposable income relative to the high cost of food is exposed to food insecurity. Although adopting healthy dietary habits may be associated with long-term savings on healthcare spending [19], financial efforts required to fully adhere to a healthy dietary pattern, such as the Mediterranean diet, may be prohibitive for some patients with a low disposable income relative to high-cost foods (e.g., fresh seafood) [20]. Personalisation also means compromising on what foods the patient can afford.

### 3.10. Society

Elements of social nature are a broad range of factors concerning family or caregiver roles, such as parental education or assistance during eating, and other environmental determinants associated with nutrition. Examples are the definition of who goes grocery shopping and when, the proximity of the grocery store, the preference to buy online, or planning based on the need for fresh or long shelf-life foods that can be stocked in the kitchen. Social elements related to food delivery have been critical during the lockdown for the pandemic of the coronavirus disease of 2019. Other non-modifiable social elements may be war, illiteracy, civil disorders, and underdevelopment [7]. Additionally, the nutritional intervention can be personalised based on the food products’ sustainability to pursue the global need to reduce the burden of greenhouse gas emissions and water use [21].

### 3.11. Counseling

Subclinical conditions of stress and anxiety, the lack of an opposition stage of the patient’s readiness for dietary change (pre-contemplation, contemplation, preparation, action, maintenance, relapse, termination), the presence of the cognitive bias known as the Dunning–Kruger effect ([22]), or unrealistic expectations regarding the nutritional goals are examples of elements with great impact on the adherence and success of the nutritional therapy. Similarly, it may be preferred to split the nutritional intervention, for example, the educational material, into more than one visit to measure out the advice and let the information be absorbed through multiple reiterations, rephrasing, and reframing. The personalised counselling has the scope of promoting patient activation (i.e., to train an individual who is able to maintain the dietary behaviour and push further) [23].

### 3.12. Reporting and Contact

Preparing the way how information relating to nutritional indications is reported to the patient based on the patient’s needs is important to improve patient experience. There may be individuals with special needs (e.g., those visually impaired) and different education levels, or there may be a language barrier between the healthcare professional and the patient. The use of personalised paper or digital patient booklets, infographics, or other tools like virtual reality [24] can help to paint a vivid picture of what patients are expected to know and do in practice. Tele-health has the potential to make nutrition care more accessible, providing instant communication channels and clinical services at home. Synchronous and asynchronous contacts through mobile apps or web portals, videos, and games may be all valuable non-inferior options, which can help also reduce the per capita cost of health care [25].

### 3.13. Food Composition Databases

The nutritional intervention can translate into a diet therapy plan generated after consulting bromatological databases, which gives information to the clinical dietitian on the amount of nutrients contained in different foods. These data are also used to establish reference values for recommendations. Diverse global repositories exist (e.g., International Network of Food Data Systems, INFOODS, by the Food and Agriculture Organisation, FAO [26]) and are constantly updated to include novel foods and reflect modern agricultural practices, climate change effects, and food processing methods. A judicious choice of what type of database to consult is a critical element to personalise foods and recipes on the region of origin and to deliver an intervention based on high-quality data.

### 3.14. Genotyping

Advances in genome-wide single nucleotide polymorphism (SNP) data collection, which concern how different allele frequencies can impact the same biological pathway, will help explain how a patient’s genetic variation in some nutrient-sensitive genes can influence the metabolic response to a diet [27]. Examples are the different regulations of caffeine sensitivity, alcohol dependence, or appetite in individuals with an excess of fat deposits [28]. While the field is still emerging and it is not yet ready because of the lack of well-designed clinical studies, future nutrition principles may be personalised based on the individual differences in metabolic response to foods.

### 3.15. Gut Microbiota

The community of microorganisms (bacteria, viruses, fungi, and protozoa) living in the gastrointestinal tract is a virtual living organ of the human body that serves many functions [29], including that of modulating nutrient bioavailability. Studies have shown that microbiome composition can influence the magnitude of postprandial lipaemia more than the macronutrient composition of the meal [30], independently predict glycaemic response to bread [31], or even modulate therapeutic outcomes of diseases through modification of drug pharmacokinetics [32]. Similarly to the genotyping element, gut microbiome testing to personalise the diet is still in its infancy, and more advancements are required to address consistency of results and overcome the costly technologies.

### 3.16. Gender Identity and Sexual Behaviours

Risk thresholds of nutrition indicators (e.g., waist-to-hip ratio, normal ranges of haemoglobin) and equations to estimate basal needs are sex-specific. Gender is conceptually distinct from sex as it refers not to the biological characteristics but to socio-cultural attitudes, identity, and orientation. Understanding the role of gender on the nutritional parameters might unveil important differences in terms of food intake, speed of eating [33], metabolic needs, nutrient-sensitive gene expression, and diet therapy either in the biological or in the socio-cultural domain [34]. Future research will have to elucidate any differences underlying gender-based nutrition.

## 4. Conclusions

**Figure 1:**
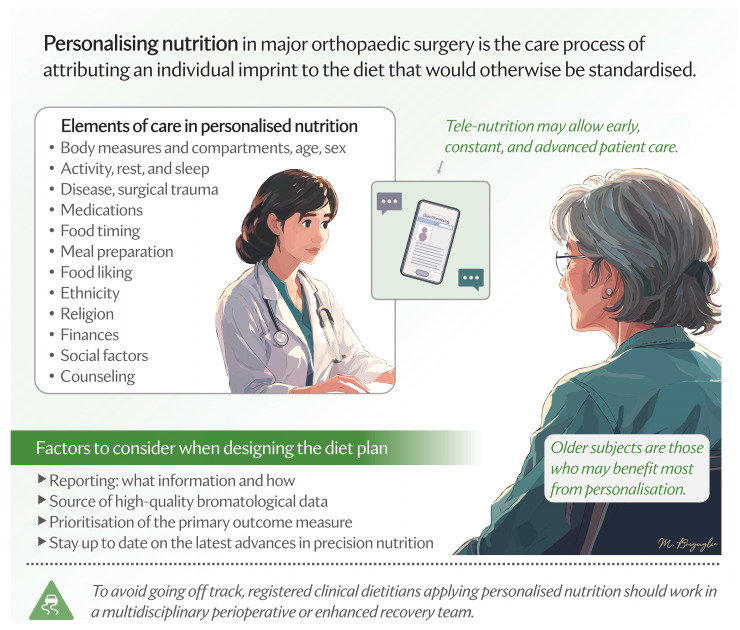
Elements of care process in the personalisation of nutrition in major orthopaedic surgery.

Personalisation represents a natural evolution of the enhanced recovery framework. Enhanced recovery provides a “standardised” model, and personalisation can refine it to the multifaceted elements of nutrition care, offering the potential to further optimise care for those with specific nutritional needs, such as older adults (Figure 1).

Given the multitude of elements linked to the individual nutritional sphere, it is reasonable to think that the introduction of personalised nutrition care into clinical practice carries with it a considerable workload for the healthcare professional. A solution could be the use of artificial intelligence (AI) technologies as a means to maintain efficiency, with AI-driven tools that can support early clinical diagnostics of patients at risk of malnutrition or already malnourished, personalise treatment recommendations, dynamically adapt the intervention, optimise meal planning in real time, and assist in clinical documentation. While these AI innovations hold promise, their misuse could risk de-professionalising healthcare and undermining patient trust, and there are logistical, cost, and governance issues to consider [35].

Furthermore, the benefit of personalised nutrition is not proven yet [2]. To make that possible, four key practical directions should be addressed in the near future. First, there is the need for criteria that identify patients who would benefit the most from personalised nutrition. Second, a roadmap for clinical studies in the nutritional field applied to major orthopaedic surgery should prioritise participation of all ages, races, and genders; control for as many of the abovementioned elements of personalisation as possible, including long-term monitoring of social, cultural, and economic factors; and explore effectiveness of diverse forms and products for nutritional care and therapy, such as disease-specific therapeutic diets, oral nutritional supplements, and other forms of administration other than oral (enteral and parenteral). Third, the elements on which to personalise nutrition are several, from the medical and pharmacological aspects to the more environment-related factors. What level of personalisation do we need to reach to have the desired effect? What are the necessary elements to consider beyond which the advantage gained in terms of benefits does not justify further personalisation? Fourth, how will care providers address situations where patients are resistant to modifications in their diet? Initiatives, such as dietitian-designed meals, medically tailored groceries, or produce prescriptions, can help overcome the difficulty of integrating dietary prescriptions into daily practice [36]. However, achieving equitable access to personalised nutrition requires integration into publicly funded healthcare systems, where comprehensive insurance coverage could support patients in receiving customised dietary care.

Standardisation has undeniably improved care quality, ensuring consistent outcomes for many orthopaedic patients who share similar post-operative needs. Yet, personalisation may add an additional layer of quality for those with distinct or unique requirements. It has the potential to avoid inefficiencies in treating patients who fall outside standard protocols, ensuring more effective and equitable care. Delaying the integration of personalised nutrition risks perpetuating healthcare inequalities, leaving some patients marginalised and underserved.

## Figures and Tables

**Table 1:** Glossary of term used when referring to a personalisation of the diet.

Approach	Definition
Individualised nutrition	Action or process of conferring an individual’s own needs to the intervention or, in other words, to render them appropriate to an individual.
Tailored nutrition	The term derives from the tailor’s work, where a garment is suited to fit the subject’s measurements and preferences. It considers a pre-existing dress code.
Optimal nutrition	It refers to the provision of the best, most favourable, and possible treatment based on a set of circumstances, such as available resources.
Precision nutrition	It roots in the terms of cutting out, removing the superfluous, or reducing to the essential. In data science, it defines the minimisation of errors to achieve high accuracy.
Customised nutrition	The term recalls the modification of a standard service to accommodate specifications or requirements of the subject.
Personalised nutrition	Umbrella term that defines the practice of attributing a personal imprint to the dietary intervention. Unlike the term customisation, which reveals direct subject control over adjustments, personalisation is closer to the concepts of individualised and tailored since it may involve essential prerequisites set by the health professional or available resources.

**Table 2:** Glossary of terms used when referring to food–drug interactions [9].

Food–Drug Interaction	Definition
Type I	Also called pharmaceuticals, they refer to ex vivo bioinactivations usually occurring in delivery devices (e.g., tablets) with hydrolysis, oxidation, neutralisation, precipitation, or complexation reactions.
Type II	They affect the function of an enzyme (type A interactions, pharmacokinetic) or transport mechanism (type B interactions, pharmacokinetic), altering absorption and bioavailability before systemic circulation. Complexation, binding, or others may occur in the gastrointestinal tract (type C interactions, pharmaceutical).
Type III	Pharmacokinetic interactions that occur after entrance into systemic circulation, including changes in tissue distribution, penetration, or metabolism.
Type IV	Pharmacokinetic interactions that include affections in drug or food component clearance because of interferences upon renal or enterohepatic excretion.
Type V	Also called pharmacodynamics, they are the indirect result of previous pharmaceutical/pharmacokinetic interactions or of direct interactions with food molecules acting against the same drug targets and competing for target-binding.
